# One-Cycle Windowed-DFT Harmonic Estimation with Spectral-Interference Compensation

**DOI:** 10.3390/s26144362

**Published:** 2026-07-09

**Authors:** Chemseddine Allioua, Alessandro Mingotti, Roberto Tinarelli, Lorenzo Peretto

**Affiliations:** 1 Independent Researcher, 37134 Verona, Italy; 2 Department of Electrical, Electronic and Information Engineering, University of Bologna, 40136 Bologna, Italy; roberto.tinarelli@unibo.it (R.T.); lorenzo.peretto@unibo.it (L.P.)

**Keywords:** harmonic estimation, spectral interference, windowed DFT, power quality, one-cycle analysis, Tikhonov regularization, frequency tracking

## Abstract

Accurate harmonic estimation at the per-cycle timescale is increasingly required in modern power-quality (PQ) monitoring, where fast-varying distortion sources demand high temporal resolution. However, when harmonic phasors are estimated from a single cycle using windowed discrete Fourier transform techniques, off-nominal fundamental frequency introduces spectral interference between harmonics, leading to systematic amplitude and phase errors that conventional correction methods cannot remove. This paper presents a lightweight, non-iterative harmonic estimation module designed to operate on fixed-rate, one-cycle data streams. The method leverages a frequency estimate provided by an external tracker to explicitly model the spectral interference induced by windowing under off-nominal conditions. By formulating this effect as a linear mixing process, the proposed approach applies an algebraic inversion to recover unbiased harmonic phasors without requiring adaptive resampling, variable window lengths, or modifications to the acquisition system. The module is designed as a plugin component compatible with existing PQ processing chains and shared sampled-value architectures. Experimental validation across frequency sweeps, Monte Carlo noise trials, and dynamic streaming scenarios demonstrates machine-precision accuracy in ideal conditions and noise-limited performance in realistic settings. Compared to iterative alternatives, the proposed solution achieves equivalent accuracy with a 484× reduction in computation time. A sensitivity analysis further quantifies the relationship between frequency-tracking accuracy and harmonic estimation error, providing practical guidelines for system integration. These results show that accurate, real-time harmonic estimation can be achieved from single-cycle data using fixed-rate acquisition, enabling improved monitoring and protection capabilities in modern power systems.

## 1. Introduction

Real-time power-quality (PQ) monitoring increasingly demands harmonic information at the per-cycle time scale because modern distortion sources are non-stationary at exactly that scale. Power-electronic loads (e.g., Electric vehicle fast chargers, variable-speed drives, and converter-interfaced generation) can change harmonic content from one cycle to the next due to control actions, operating-point transitions, and grid interactions. PQ tasks such as event-triggered diagnosis, fast harmonic-resonance supervision, and short-time compliance assessment therefore require cycle-by-cycle visibility rather than multi-cycle averages [1,2,3]. Multi-cycle processing smooths these dynamics and can mask short but operationally critical excursions. The same requirement appears in protection applications, where resonance-driven or converter-driven harmonic transients must be identified within one or two cycles to trigger timely protective actions.

The standard approach in PQ instruments is to apply a windowed DFT and compensate the window’s gain. However, when only one cycle of data is available, the DFT bin spacing equals the harmonic spacing: under off-nominal fundamental frequency, tones miss their bin centers and the window’s spectral response couples adjacent harmonics into every bin [4]. This effect, referred to here as spectral interference (i.e., leakage-induced coupling between harmonics), produces systematic amplitude and phase bias that no amount of gain correction can remove.

Classical leakage-mitigation strategies solve off-nominal operation by adapting the acquisition itself (dynamic resampling or frequency-locked variable windows). In modern digital substations, however, acquisition is usually centralized in a merging unit that publishes one shared sampled-value stream to multiple IEDs (PQ analyzers, protection relays, PMUs, and others). In this architecture, the sampling system cannot be tailored dynamically for only one downstream function: the sampling rate is typically fixed for all clients. With fixed sampling, off-nominal frequency inevitably produces spectral interference in one-cycle DFT-based harmonic estimation.

Despite extensive work on harmonic estimation, there is currently no method that simultaneously operates on one-cycle, fixed-rate data, remains compatible with shared acquisition architectures, and provides accurate multi-harmonic estimates under off-nominal frequency without iterative processing. Existing solutions either require adaptive acquisition, rely on per-harmonic filtering, or do not explicitly address joint multi-harmonic interference.

Intuitively, the proposed approach treats spectral interference not as random error but as a deterministic coupling between harmonics induced by the windowed DFT, which can be explicitly modeled and inverted.

This paper proposes a lightweight module that takes a tracker-provided frequency estimate ${\hat{f}}_{1}$ and corrects that leakage algebraically on top of the fixed-rate data stream. To avoid a common misreading, the scope is stated precisely: the fundamental frequency is an input supplied by an external tracker—it is not estimated from the windowed DFT—and the module recovers the harmonic phasors (amplitudes and phases) by inverting the deterministic, frequency-dependent mixing that windowing induces. We do not claim that gain-corrected windowing alone removes leakage; on the contrary, Section 5 shows that it cannot, which is precisely why the inversion is needed. Crucially, the correction operates on a fixed-length DFT frame: no adaptive resampling, no variable window length, and no change in sampling rate are required. The module is therefore compatible with shared acquisition infrastructures, fixed-point hardware, radix-2 FFT engines, and existing buffer management.

### 1.1. Related Work

Harmonic content estimation is central to PQ monitoring standards [2,3]. The per-cycle setting addressed here is a specific high-latency-constrained operating case. For this scoped problem—harmonic estimation from one-cycle, fixed-rate data under off-nominal frequency—prior work is best organized into two directly relevant families, plus one adjacent line of research.

Windowed and interpolated-DFT (IpDFT) lineage. DFT-based estimation of sinusoidal parameters has matured over nearly two decades, and windowed-DFT is only one category within it. Single-tone interpolation was established early [5,6,7] and progressively refined through Fourier-coefficient and iterative interpolators [8,9,10,11]; multipoint and cosine-window variants subsequently improved robustness to harmonics and off-nominal frequency [12,13,14,15], and FPGA/edge realizations brought these estimators to real-time deployment [16,17]. Beyond the windowed/IpDFT family, the broader landscape includes subspace and parametric estimators (ESPRIT, matrix pencil, Prony) [18,19,20] and sparse/compressive formulations [21]. The present work belongs to the windowed-DFT category but departs from the prevailing single-tone, per-harmonic interpolation paradigm: rather than localizing each tone independently, it models the interharmonic coupling jointly and inverts it in closed form. The remaining gap for the per-cycle, fixed-rate regime is detailed next.

Acquisition-adaptive methods reduce leakage by adapting the sampling process (dynamic resampling or frequency-locked windowing) [22,23]. These approaches can be effective, but they require tight coupling between frequency tracking and acquisition, which is difficult to deploy in shared sampled-value architectures.

Per-harmonic filtering methods (e.g., SOGI/DIQ-based approaches) attenuate interference in the time domain before phasor extraction [24,25]. They require one adaptive filter path per harmonic and introduce tuning/settling trade-offs under fast dynamics.

Adjacent line (fundamental-focused iterative subtraction): Methods such as FiIpDFT and related extensions [26,27] estimate and subtract spectral interference primarily for fundamental phasor estimation, not full multi-harmonic extraction. They are therefore informative from a leakage-cancellation perspective but are not direct solutions to the target problem addressed in this paper.

General spectral-estimation families such as sparse/compressive models or parametric multi-tone techniques [18,21,28] are powerful in broader settings, yet they typically rely on multi-cycle data, sparsity priors, or higher computational complexity than practical per-cycle PQ pipelines. Harris [29] provides foundational window-analysis context.

Within this scoped problem, existing methods either require adaptive acquisition, rely on per-harmonic filtering, or do not address joint multi-harmonic interference. A method that combines fixed-rate operation, non-iterative processing, and joint harmonic recovery remains unavailable.

### 1.2. Contributions

The proposed approach assumes integer harmonics, negligible interharmonic content, and the availability of a fundamental-frequency estimate from an external tracker. Within these assumptions, it enables accurate per-cycle harmonic estimation while remaining compatible with existing PQ processing pipelines.

- A one-cycle spectral-interference model that describes leakage-induced coupling between harmonics under off-nominal frequency conditions.
- A non-iterative harmonic estimation module that inverts this coupling through a regularized linear system, enabling accurate joint recovery of harmonic phasors from fixed-rate data.
- A sensitivity analysis that maps tracker accuracy to harmonic estimation error, providing a practical specification for pairing the module with existing frequency trackers.
- Comprehensive experimental validation—including noiseless sweeps, Monte Carlo noise trials, frequency-error sensitivity, and a 60 s streaming scenario—comparing the module against four baselines that span plain DFT, interpolated DFT, and iterative spectral subtraction.

This capability enables accurate real-time harmonic monitoring in modern power systems without requiring modifications to existing acquisition infrastructures.

### 1.3. Paper Organization

The remainder of this paper is organized as follows. Section 2 analyzes the effect of windowing in one-cycle DFT-based harmonic estimation and highlights the spectral-interference problem. Section 3 introduces the proposed compensation method and its algebraic formulation. Section 4 discusses window-selection trade-offs and conditioning effects. Section 5 presents the baseline and competing methods used for comparison. Section 6 and Section 7 evaluate the proposed module under ideal and noisy conditions, respectively, while Section 8 analyzes sensitivity to frequency-estimation errors and derives practical tracker requirements. Section 9 provides end-to-end validation of the complete tracker-plus-module pipeline. Section 10 discusses implementation aspects and computational cost, Section 11 summarizes practical implications and limitations, and Section 12 concludes the paper.

## 2. Effect of Windowing in a One-Cycle Frame

Let the sampled signal over one frame be

(1) $$x\left[n\right]=\sum_{m=1}^{K}H_{m}\;e^{j2\pi \frac{mf_{1}}{f_{s}}n}+\eta \left[n\right],\;n=0,\ldots ,N-1,$$

with sampling rate $f_{s}$, nominal fundamental $f_{0}$, actual fundamental $f_{1}=f_{0}+\delta f$, and $N=\frac{f_{s}}{f_{0}}$ (exactly one nominal cycle). The complex phasor $H_{m}\in C$ encodes amplitude and phase of the *m*-th harmonic. Noise η[n] is optional. Define the one-cycle DFT:

(2) $$X\left[k\right]=\sum_{n=0}^{N-1}x\left[n\right]\;w\left[n\right]\;e^{-j2\pi \frac{k}{N}n},\;k=0,\ldots ,N-1,$$

where w[n] is a finite-length window function. The rectangular window corresponds to w[n]≡1, whereas cosine-sum windows (e.g., Hamming, Blackman–Harris) are designed to control sidelobe levels.

For Hamming,

(3) $$w\left[n\right]=a_{0}-a_{1}\cos\;\left(\frac{2\pi n}{N}\right),\;a_{0}=0.54,\;a_{1}=0.46.$$

Because harmonic *m* sits at *fractional* bin position m(1+ρ), with

(4) $$\Delta f=\frac{f_{s}}{N}=f_{0},\;\rho ≜\frac{\delta f}{\Delta f}=\frac{f_{1}-f_{0}}{f_{0}},$$

its windowed contribution to bin *k* equals the window DTFT evaluated at the bin offset:

(5) $$X_{k}^{\left(m\right)}=H_{m}\;W\;\left(k-m(1+\rho )\right).$$

The window’s DTFT, for cosine-sum windows, is expressed as a finite sum of shifted Dirichlet kernels:

(6) $$\begin{array}{cc} W(\nu ) & =\sum_{r\in R}c_{r}\;D_{N}\left(\nu -r\right), \end{array}$$

(7) $$\begin{array}{cc} D_{N}\left(\nu \right) & =e^{-j\pi \nu \frac{N-1}{N}}\frac{\sin(\pi \nu )}{\sin(\pi \nu /N)}. \end{array}$$

Using Equation (7), Equation (5) becomes

(8) $$X_{k}^{\left(m\right)}=H_{m}\sum_{r\in R}c_{r}\;D_{N}\;\left(k-m(1+\rho )-r\right).$$

For the rectangular window, R={0}; for the Hamming window, R={−1,0,+1} with coefficients $c_{0}=0.54$ and $c_{\pm 1}=-0.23$; and for the Blackman–Harris (4-term) window, R={−3,…,+3} with tabulated coefficients [4,29,30].

Stacking all harmonics yields the measured bin composition:

(9) $$X_{k}=\sum_{m=1}^{K}\sum_{r\in R}H_{m}\;c_{r}\;D_{N}\;\left(k-m(1+\rho )-r\right).$$

Equation (9) highlights two fundamental windowing effects in one-cycle analysis:

1. Sidelobe control (leakage mitigation): The rectangular DTFT has a first sidelobe around −13dB, while Hamming reduces it to −43dB and the 4-term Blackman–Harris to about −90dB. Lower sidelobes minimize energy spreading from distant harmonics.
2. Main-lobe widening (spectral interference): In a one-cycle frame, the DFT bin spacing is $\Delta f=f_{0}$—the same as the harmonic spacing. Under off-nominal operation (ρ≠0), window main lobes centered at m(1+ρ) overlap neighboring harmonics. Cosine-sum windows widen the main lobe, reducing distant leakage but increasing local spectral interference.

It is essential to note that every term in Equation (9) is deterministic and fully known once ρ is given: the cosine-sum replicas at offsets r∈R—i.e., the additional components a window introduces near $\omega \pm \frac{2\pi }{N}$—are not a stochastic leakage error but a fixed linear map from the harmonic phasors to the measured bins. The proposed method (Section 3) therefore does not attempt to suppress this interference with a better window or to read frequency off the windowed spectrum; instead, it inverts the known map to recover the phasors, turning what is conventionally treated as an error source into the forward model of an estimator.

**Figure 1:**
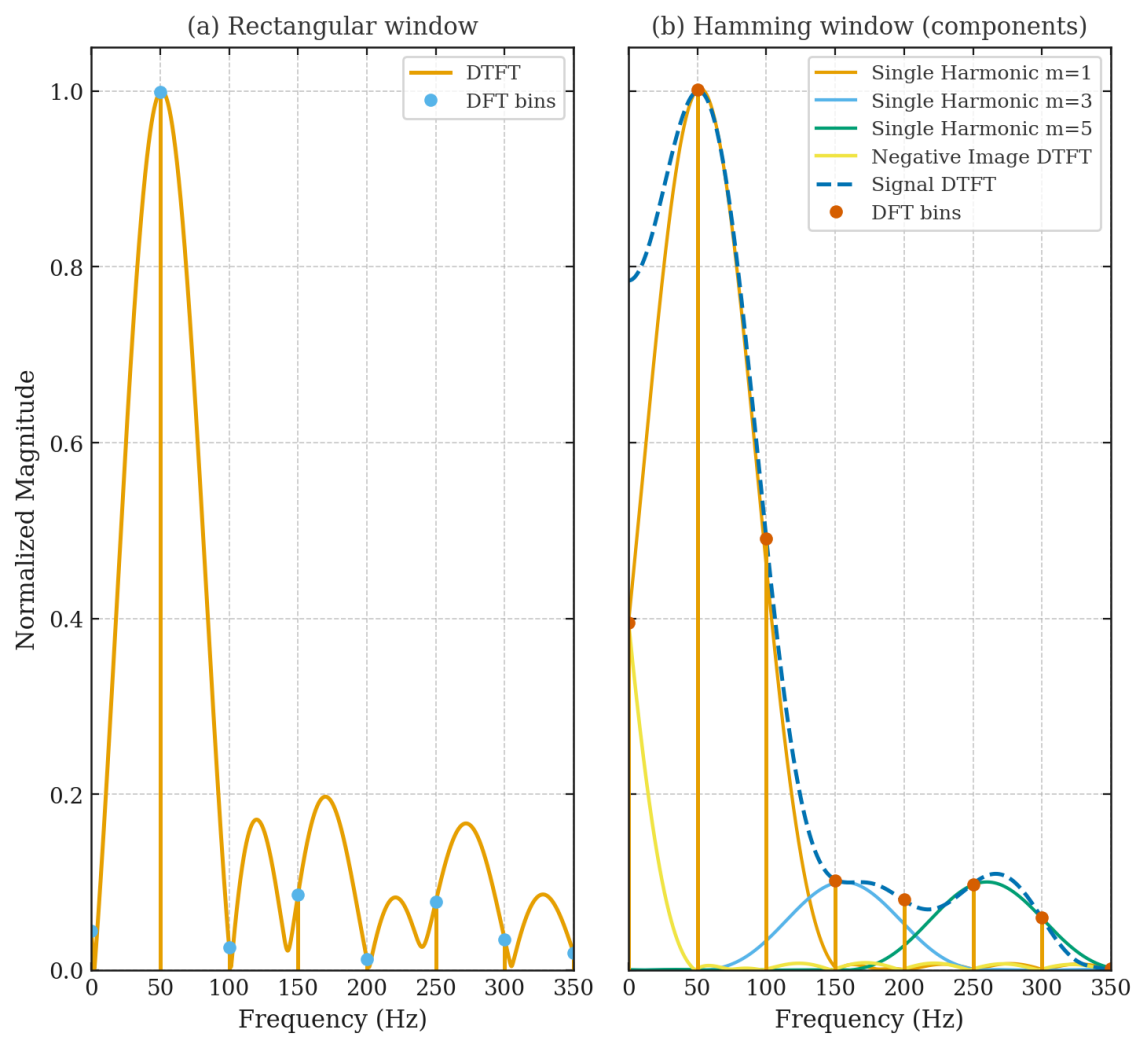
Fourier Transform analysis over one nominal cycle ($N=f_{s}/f_{0}$). (**a**) Rectangular window: DTFT (blue) and DFT bins (orange) show strong leakage and off-bin sampling. (**b**) Hamming window: sidelobes are reduced, but main-lobe widening increases overlap between harmonics; thin curves show single-harmonic DTFTs. Frequency axis in hertz.

Figure 1 illustrates these effects for a signal composed of the fundamental plus the 3rd and 5th harmonics (each at 10% amplitude), with $f_{1}=52\;\mathrm{Hz}$, $f_{0}=50\;\mathrm{Hz}$, and $N\;=\;f_{s}/f_{0}\;=\;128$ samples. When the rectangular window is applied: the DTFT (blue) shows narrow lobes but high sidelobes, and the DFT bins (orange markers) miss the tone peaks due to the fractional-bin offset, resulting in leakage into adjacent bins. On the other hand, the Hamming window suppresses sidelobes, but its wider main lobe causes overlap between harmonics. Each thin curve corresponds to a single harmonic’s DTFT, showing that individual lobes intersect near the DFT bin centers, which explains amplitude and phase bias in standard DFT readings.

The consequences of this coupling for different estimation strategies are explored in Section 5.

## 3. Spectral-Interference Compensation

This section proposes a solution for the previously described spectral interference while windowing a one-cycle frame under off-nominal frequency conditions. The central idea is to explicitly represent the spectral interference between harmonics as known linear mixing governed by the discrete-time Fourier transform (DTFT). Instead of treating spectral leakage as a random error, we model it analytically through the window’s replica expansion. The assumptions made to ensure that the technique works are that (i) the actual fundamental frequency $f_{1}$ (or its normalized offset $\rho =\left(f_{1}-f_{0}\right)/f_{0}$) is known or tracked for each frame; (ii) only integer harmonics of order up to *K* are relevant, with negligible interharmonics; and (iii) the sampling frequency is sufficiently high to avoid aliasing.

### 3.1. The Linear System X=W(ρ)H

Equation (9) shows that the measured bin $X_{k}$ is the linear sum of contributions from all harmonics, each filtered by the window DTFT, which is itself a sum of shifted Dirichlets. The off-nominal frequency appears through ρ, shifting the center of the main lobe of each harmonic.

Collect bins k=1,…,K (first *K* positive-frequency bins) into $X\in C^{K}$. Define $H={\left[H_{1},\ldots ,H_{K}\right]}^{T}\in C^{K}$. From Equation (9),

(10) $$\begin{array}{cc} X_{k} & =\sum_{m=1}^{K}W_{k,m}\left(\rho \right)\;H_{m}, \end{array}$$

(11) $$\begin{array}{cc} W_{k,m}\left(\rho \right) & ≜\sum_{r\in R}c_{r}\;D_{N}\;\left(k-m(1+\rho )-r\right). \end{array}$$

In matrix form,

(12) X=W(ρ)H.

Here, $W\left(\rho \right)\in C^{K\times K}$ describes the deterministic cross-talk between harmonics introduced by the finite window. For cosine-sum windows such as Hamming or Blackman–Harris, W(ρ) is *banded*: only a few neighboring harmonics around each bin contribute significantly, with the bandwidth dictated by the main-lobe width of the window’s DTFT. In the case of the Hamming window, meaningful coupling typically involves harmonics m∈{k−1,k,k+1}.

**Figure 2:**
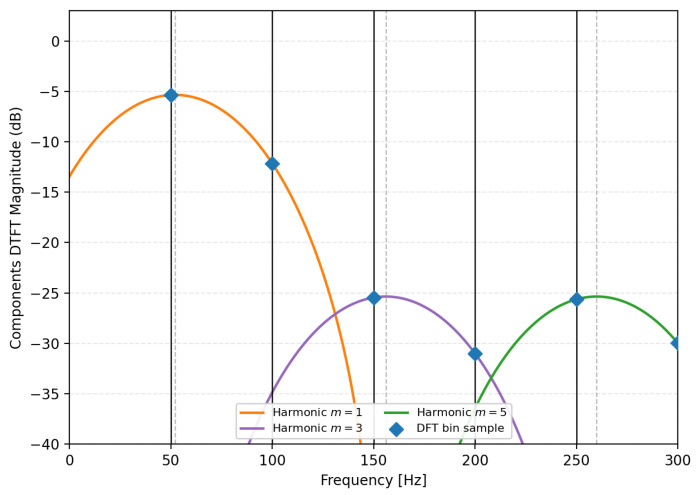
Distribution of DFT magnitude for Hamming main lobes at harmonic frequencies.

Figure 2 shows the Hamming main lobes for each harmonic of the signal used in Figure 1. Each lobe is centered on its corresponding harmonic frequency, marked by dashed lines. The blue diamonds indicate the primary spectral contributions to the DFT bin at these frequencies.

### 3.2. Tikhonov-Regularized Inversion

The harmonic vector *H* can then be estimated by solving a regularized least-squares problem:

(13) $$\hat{H}=\underset{H}{arg min}\;∥W\left(\rho \right)H-X{∥}_{2}^{2}+\lambda {∥H∥}_{2}^{2},$$

which admits the analytical Tikhonov solution [31]

(14) $$\hat{H}={\left(W^{H}W+\lambda I\right)}^{-1}W^{H}X.$$

A small regularization term λ stabilizes the inversion when $W^{H}W$ becomes ill-conditioned (e.g., for windows with very wide main lobes or when higher-order harmonics are included). Because W(ρ) is narrow-banded and quasi-Toeplitz, the computational burden is minimal: the inversion involves only local couplings between neighboring harmonics. Under the previously mentioned assumptions, *W* is typically full column rank; with mild λ, Equation (14) is well posed. Identifiability improves if the number of measurement bins exceeds *K* (overdetermined variants are possible, e.g., by including bins k>K or stacking overlapping frames).

Equation (12) transforms the problem from a spectral-resolution limitation into a well-defined linear algebra problem. The off-nominal deviation ρ simply shifts the Dirichlet replicas within W(ρ), and by solving Equation (14), one can reconstruct unbiased harmonic amplitudes and phases per frame.

Since W(ρ) depends only on ρ and the window function—not on the measured signal—it can be precomputed and cached whenever ρ changes slowly. In such cases, the per-frame cost reduces to a single K×K linear solve, making the method particularly attractive for real-time systems where the fundamental frequency drifts gradually.

As highlighted in Section 1, the module operates on fixed-rate, fixed-length acquisitions and therefore avoids adaptive windowing or frequency-dependent resampling. Here, this follows directly from the model: the off-nominal deviation $\rho =\left(f_{1}-f_{0}\right)/f_{0}$ is handled analytically inside X=W(ρ)H, so the window length remains fixed—typically $N=f_{s}/f_{0}$—and efficient radix-2 FFT implementations can be retained. This property is crucial for real-time and embedded systems, where timing determinism and fixed buffer sizes are mandatory.

## 4. Window Selection and Conditioning Trade-Offs

The choice of window function directly determines the structure of W(ρ) and, consequently, the numerical conditioning of the harmonic estimation problem. Each cosine-sum window defines a replica set R and coefficients $c_{r}$; wider main lobes suppress distant sidelobes but increase coupling between neighboring harmonics, enlarging the off-diagonal energy of W(ρ).

Table 1 summarizes the key properties of five standard windows. The replica count grows from 1 (rectangular) to 7 (BH4), and sidelobe levels decrease from −13 dB to −92 dB. However, the mean condition number $\kappa (W^{H}W)$ averaged over ρ∈[−0.1,+0.1] increases by orders of magnitude.

### 4.1. Conditioning Versus Frequency Deviation

**Figure 3:**
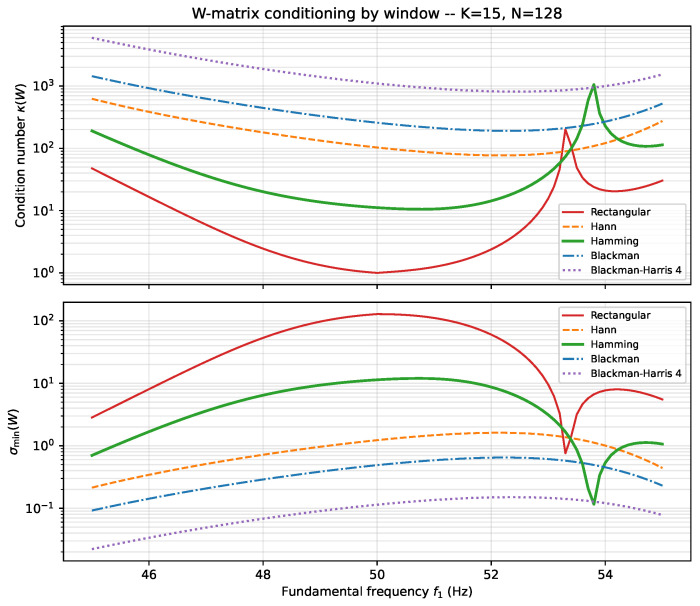
Condition number κ(W) versus fundamental frequency for five windows (K=15, N=128). A spike appears near 53.8 Hz due to harmonic-to-bin crossings.

Figure 3 plots κ(W) as a function of $f_{1}$ across the 45–55 Hz range. All windows exhibit a conditioning spike near $f_{1}\approx 53.8\;\mathrm{Hz}$ (ρ≈0.075), where harmonics $H_{13}$ and $H_{14}$ simultaneously approach neighboring integer bins, causing two rows of W(ρ) to become nearly linearly dependent. The Hamming window reaches κ≈1055 at the spike but maintains κ≈11 at nominal frequency. In contrast, the Blackman–Harris window has κ>900 even at nominal.

### 4.2. Accuracy Implications

**Figure 4:**
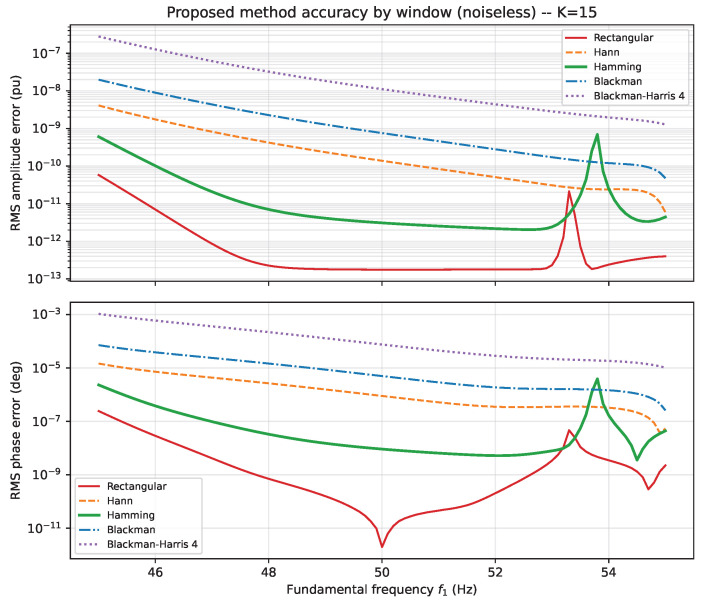
RMS amplitude error across the frequency sweep for the proposed compensated estimator with each window. All achieve near-machine precision in noiseless conditions.

Despite the conditioning variation, all five windows achieve machine-precision accuracy in the noiseless case (Figure 4). The RMS amplitude error ranges from $3.4\times 10^{-12}$ pu (rectangular) to $4.0\times 10^{-8}$ pu (BH4). Under noise, however, estimation variance scales with $\kappa^{2}$: higher conditioning amplifies measurement noise into the recovered harmonics. This creates a practical trade-off: wider windows (BH4, Blackman) offer superior sidelobe rejection for out-of-model content but incur a noise penalty through elevated conditioning.

### 4.3. Hann Versus Hamming

A noteworthy finding is that Hann exhibits significantly higher conditioning than Hamming at nominal frequency (κ=103 vs. κ=11), despite both using three replicas. This arises from Hann’s symmetric cosine coefficients ($c_{0}=0.5$, $c_{\pm 1}=-0.25$), which create a wider effective main lobe and stronger inter-bin coupling than Hamming’s asymmetric coefficients ($c_{0}=0.54$, $c_{\pm 1}=-0.23$).

### 4.4. Recommendation

For general per-cycle PQ estimation, the Hamming window offers the best balance: a narrow replica set (R={−1,0,+1}), low conditioning (κ≈11 at nominal), and −43 dB sidelobe suppression. The rectangular window can outperform it in clean, well-filtered signals due to its unit condition number, but is fragile to out-of-band content. Blackman and BH4 are preferable only when very low sidelobes are essential and the system is overdetermined or stronger regularization is acceptable. All subsequent experiments in this paper use the Hamming window for the proposed and iterative estimators.

## 5. Baseline and Competing Methods

To assess the proposed spectral-interference compensation module, we compare against four reference methods. All methods receive the same one-cycle frame of $N=f_{s}/f_{0}$ samples and produce *K* harmonic phasor estimates ${\hat{H}}_{1},\ldots ,{\hat{H}}_{K}$. To avoid methodological ambiguity, we separate them into: (i) external practical baselines widely used in fixed-rate PQ pipelines, and (ii) adapted/in-house comparators used to probe specific algorithmic effects.

### 5.1. Group A: External Practical Baselines

These methods represent common fixed-rate PQ practice and do not attempt explicit interharmonic unmixing.

#### 5.1.1. Method 1: Rectangular DFT

The simplest baseline applies no window and reads each DFT bin directly:

(15) $${\hat{H}}_{m}=\frac{1}{N}\sum_{n=0}^{N-1}x\left[n\right]\;e^{-j2\pi mn/N}.$$

At nominal frequency (ρ=0), the rectangular DFT is exact—harmonics land on bin centers with no leakage. At ρ≠0, the sinc-like sidelobes (−13 dB first sidelobe) cause cross-talk from all harmonics into every bin. This baseline serves as the “do nothing” reference under the fixed-*N* constraint.

#### 5.1.2. Method 2: Hamming Gain-Corrected DFT

Standard practice in PQ instruments is to apply a window and divide by its DC gain:

(16) $${\hat{H}}_{m}=\frac{X_{m}}{\sum_{n=0}^{N-1}w\left[n\right]},$$

where $X_{m}$ is the windowed DFT at bin *m* and w[n] is the Hamming window. This corrects the overall amplitude scaling introduced by the window but ignores the interharmonic coupling described in Section 2. As shown in Section 4, the Hamming main lobe spans three bins; at ρ≠0, adjacent harmonics contribute significantly to each bin, producing systematic amplitude and phase bias.

#### 5.1.3. Method 3: Per-Harmonic IpDFT Adaptation (Hann)

Interpolated DFT (IpDFT) methods estimate the fractional-bin offset of each tone from the ratio of neighboring DFT magnitudes [6,11]. For each harmonic *m*, a Hann-windowed three-point estimator computes [7,12]

(17) $$\delta_{m}=\frac{|X_{m+1}\left|-\right|X_{m-1}|}{2|X_{m}\left|-\right|X_{m-1}\left|-\right|X_{m+1}|},$$

from which the corrected amplitude and phase follow via the window’s DTFT evaluated at $\delta_{m}$. In this paper, IpDFT is used as a per-harmonic adaptation: the same single-tone routine is repeated for each *m*, with $\hat{\rho }$ used only for bin localization. This approach is effective for isolated tones, but in a one-cycle frame, the bins m±1 contain energy from harmonics m±1—not just the sidelobes of harmonic *m*—so the isolated-tone assumption breaks down [32]. The Hann window is used per IpDFT convention [12].

### 5.2. Group B: Adapted/In-House Comparators

These methods use $\hat{\rho }$ and are included to separate algorithmic effects within the same fixed-rate framework.

#### 5.2.1. Method 4: Iterative *W*-Model Solver (Hamming)

This is an in-house iterative solver built on the same W(ρ)-coupling model used by the proposed method. In each iteration, we carry out the following:

1. Estimate all harmonics via gain-corrected Hamming DFT (Method 2).
2. For each harmonic *m*, subtract the spectral contributions of all other harmonics from the windowed DFT bins using the Dirichlet replica model.
3. Re-estimate each harmonic from the cleaned bins.

This process is repeated for up to 20 iterations, retaining the iterate with the smallest residual ${∥X-W\left(\hat{\rho }\right)\hat{H}∥}_{2}$. It serves as an iterative counterpart to Method 5 (same model family, different solver strategy: iterative subtraction vs one-shot inversion).

#### 5.2.2. Method 5: Proposed *W*-Matrix Module (Hamming)

The proposed add-on module builds the spectral-interference matrix W(ρ) as described in Section 3 and solves the regularized system in one shot:

(18) $$\hat{H}={\left(W^{H}W+\lambda I\right)}^{-1}W^{H}X,\;\lambda =10^{-8}.$$

The Hamming window with replicas R={−1,0,+1} is used, yielding a tridiagonal-like *W*. The module is non-iterative, deterministic, and exact for the assumed signal model. Unlike the other frequency-aware approaches, it solves all harmonics jointly in a single algebraic step, see Table 2.

## 6. Module Accuracy with Perfect Frequency Knowledge

This section isolates the intrinsic accuracy of the harmonic module by supplying the true ρ (oracle frequency). It answers the question: given a perfect tracker, how well can each method recover the harmonic phasors?

All tests used $f_{s}=6.4\;\mathrm{kHz}$, N=128, and K=15 harmonics with realistic amplitudes and phases representative of power-system distortion. The fundamental frequency was swept from 45 Hz to 55 Hz in 0.1 Hz steps, covering the full ±10% deviation range.

### 6.1. Results over Frequency Sweep

**Figure 5:**
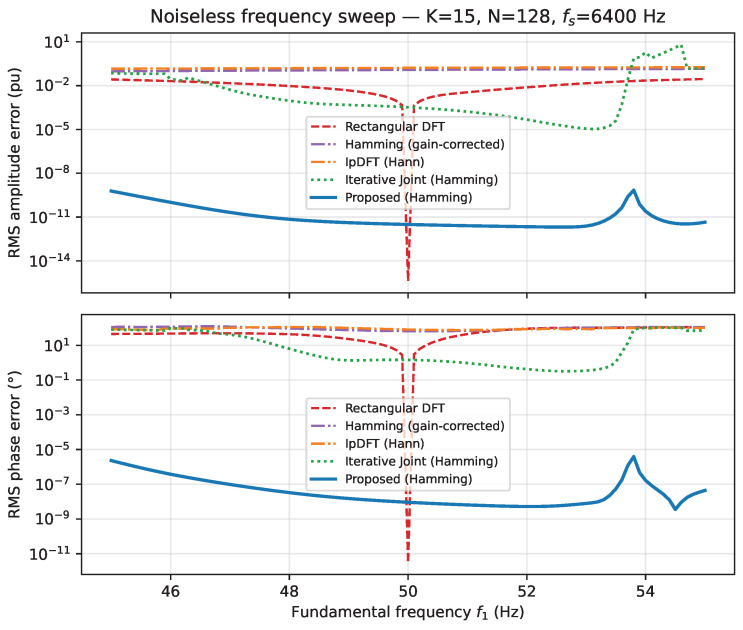
RMS amplitude and phase error versus fundamental frequency for the five methods (K=15, N=128, noiseless). The proposed module achieves machine-precision accuracy across the full 45–55 Hz range.

Figure 5 shows the RMS amplitude and phase errors as a function of $f_{1}$ for all five methods. A clear three-regime structure emerges:

- Regime 1 (strong bias): The Hamming gain-corrected and IpDFT-adaptation estimators are dominated by systematic bias, with mean RMS amplitude errors of $1.20\times 10^{-1}$ pu and $1.66\times 10^{-1}$ pu, respectively. Remarkably, the Hamming gain-corrected method performs *worse* than the unwindowed rectangular DFT: its wider three-bin main lobe introduces more interharmonic coupling than the rectangular window’s narrower sinc lobes.
- Regime 2 (moderate bias): The rectangular DFT achieves a mean amplitude error of $1.27\times 10^{-2}$ pu, limited by −13 dB sinc sidelobe leakage that grows with |ρ|.
- Regime 3 (near-exact/model-based): The proposed and iterative *W*-model methods reach machine precision near 50 Hz. However, the iterative solver diverges catastrophically near $f_{1}\approx 53.8\;\mathrm{Hz}$ (max amplitude error 6.41 pu), where the conditioning spike identified in Section 4 causes the Gauss–Seidel iteration to fail. The proposed one-shot module maintains $<10^{-9}$ pu across the full range.

Table 3 reports the maximum and mean errors across the sweep. The proposed module achieves a maximum RMS amplitude error of $6.93\times 10^{-10}$ pu—over ten orders of magnitude better than the best baseline.

### 6.2. Per-Harmonic Error Distribution

**Figure 6:**
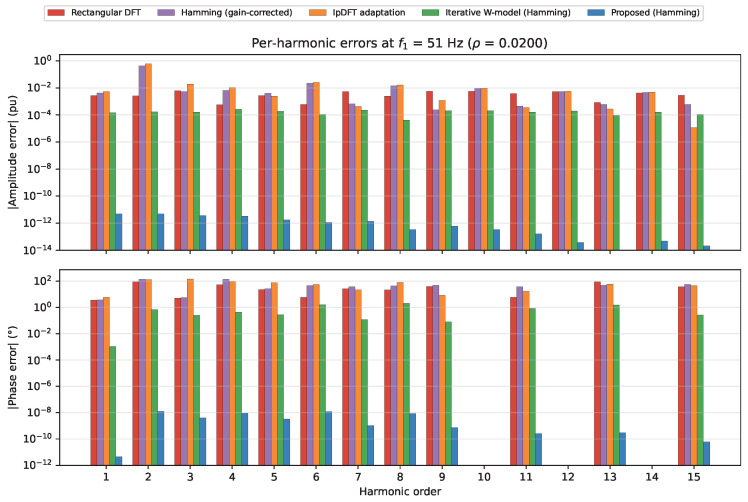
Per-harmonic amplitude and phase errors at a representative off-nominal frequency. Higher harmonics incur larger bias in the non-compensated methods.

Figure 6 shows the per-harmonic amplitude and phase errors at a representative off-nominal point. The three bias-dominated methods exhibit increasing error for higher-order harmonics, as their fractional-bin offsets grow with *m*. Methods 4 and 5 recover all harmonics to comparable accuracy, except where the iterative estimator fails at the conditioning spike.

### 6.3. Discussion

With oracle ρ, this sweep provides the intrinsic upper bound of the module. The detailed implications (bias of Group A baselines and divergence behavior of the iterative *W*-model solver) are used in Section 7, Section 8, Section 9, Section 10 and Section 11 and are therefore not repeated here.

## 7. Module Noise Floor

Section 6 established the module’s accuracy with perfect frequency knowledge and no noise. This section adds measurement noise to determine the module’s noise floor—the irreducible estimation error set by SNR when the tracker provides exact ρ.

A Monte Carlo experiment was conducted with $f_{1}=51\;\mathrm{Hz}$ (ρ=0.02), K=15, and the same harmonic profile as Section 6. Complex additive white Gaussian noise was added at SNR levels of {80, 40, 20} dB. For each SNR, 1000 independent trials were simulated, and median RMS errors were recorded.

### 7.1. Error Versus SNR

**Figure 7:**
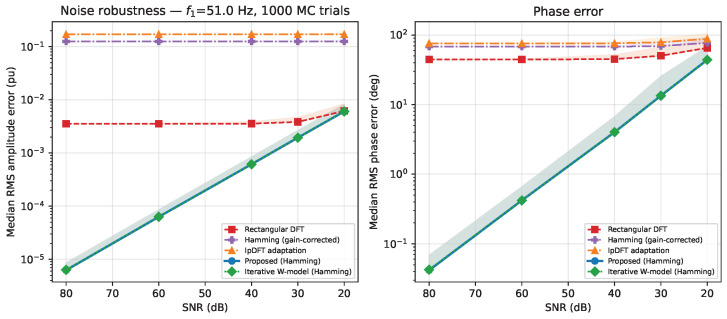
Median RMS amplitude error versus SNR ($f_{1}=51\;\mathrm{Hz}$, 1000 trials). Methods 4 and 5 track the noise floor; Methods 1–3 are bias-dominated. Shaded bands denote the median-to-95th-percentile spread across trials.

Figure 7 and Table 4 summarize the results. The three-regime structure identified in the noiseless case persists under noise:

- Methods 2 and 3 (Hamming gain-corrected and IpDFT adaptation) are bias-dominated: their errors remain virtually constant across all SNR levels (∼0.12–0.17 pu), since the systematic leakage bias far exceeds the noise floor.
- Method 1 (rectangular DFT) shows a moderate bias of ∼$3.5\times 10^{-3}$ pu that is slightly affected by noise only at 20 dB.
- Methods 4 and 5 (iterative and proposed) are noise-limited: their errors scale with the noise floor, exhibiting approximately 10× improvement per 20 dB increase in SNR, consistent with the linear scaling $\mathrm{cov}\left(\hat{H}\right)\propto \sigma^{2}$.

**Table 4:** Median RMS errors at three SNR levels ($f_{1}=51\;\mathrm{Hz}$).

Method	80 dB		40 dB		20 dB	
	Amp.	Ph.	Amp.	Ph.	Amp.	Ph.
Rect. DFT	3.54 × $10^{-3}$	44.5°	3.57 × $10^{-3}$	45.1°	6.18 × $10^{-3}$	65.4°
Hamm. gain	1.25 × $10^{-1}$	68.0°	1.25 × $10^{-1}$	68.1°	1.25 × $10^{-1}$	77.3°
IpDFT adap.	1.70 × $10^{-1}$	75.8°	1.70 × $10^{-1}$	76.1°	1.71 × $10^{-1}$	88.3°
Iterative *W*	6.34 × $10^{-6}$	0.042°	6.15 × $10^{-4}$	4.02°	6.05 × $10^{-3}$	44.1°
**Proposed**	**6.26 × 10^−6^**	**0.042**°	**6.15 × 10^−4^**	**4.02**°	**6.05 × 10^−3^**	**44.1**°

Bold indicates the proposed method (best performance).

At $f_{1}=51\;\mathrm{Hz}$, the proposed and iterative methods yield nearly identical performance across all SNR levels, both approaching the Cramér–Rao bound for multi-tone estimation [5,33]. This is expected: at moderate ρ, the iterative estimator converges to a residual of ∼$10^{-6}$ pu, which is invisible below the noise floor. The two methods separate only at extreme ρ (where the iterative diverges, cf. Section 6) or at SNR >100 dB.

### 7.2. Regularization Sensitivity

**Figure 8:**
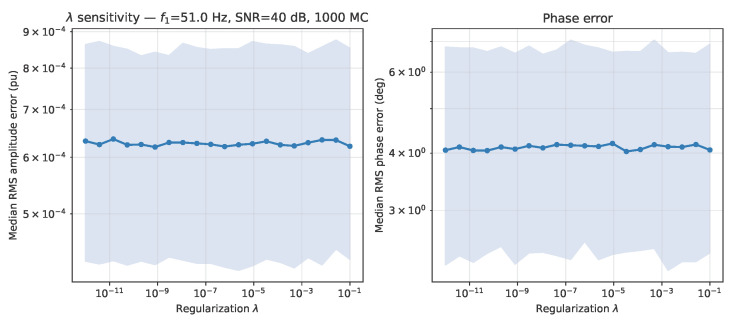
RMS amplitude error versus regularization parameter λ (40 dB SNR, $f_{1}=51\;\mathrm{Hz}$). A flat plateau over eight decades confirms insensitivity to λ in the recommended range. The shaded band denotes the 5th-to-95th-percentile spread across trials.

Figure 8 shows the effect of the regularization parameter λ on the proposed module’s accuracy at 40 dB SNR. For $\lambda \in [10^{-12},10^{-4}]$, the error is flat—the regularization is small enough not to bias the solution, and accuracy is limited by the noise floor. For $\lambda \;>\;10^{-3}$, regularization bias begins to dominate, pushing the solution toward zero. The plateau spanning eight decades of λ confirms that the method is not sensitive to this hyperparameter. We recommend $\lambda =10^{-8}$ as a robust default.

## 8. Tracker Requirements: Frequency-Error Sensitivity

The frequency-aware methods (4 and 5) require ρ from an external tracker. This section quantifies how tracker inaccuracy $\Delta \rho =\hat{\rho }-\rho_{\mathrm{true}}$ propagates into harmonic estimation error, thereby defining the minimum tracker specification for a given harmonic accuracy target. The analysis serves as a practical “spec sheet” for system integrators choosing a tracker to pair with the proposed module.

### 8.1. Theoretical Error Propagation

Three sources of error affect the Tikhonov estimator Equation (14):

#### 8.1.1. Truncation Error

If replicas beyond R are omitted, the estimation bias is

(19) $$\hat{H}-H\approx {\left({\tilde{W}}^{H}\tilde{W}+\lambda I\right)}^{-1}{\tilde{W}}^{H}\Delta W\;H,$$

where $\tilde{W}$ is the truncated model and $\Delta W=W_{\mathrm{full}}-\tilde{W}$. For Hamming with R={−1,0,+1}, this term is negligible (<−50 dB).

#### 8.1.2. Measurement Noise

Under additive noise η with variance $\sigma^{2}$,

(20) $$\mathrm{cov}\left(\hat{H}\right)\approx \sigma^{2}{\left(W^{H}W+\lambda I\right)}^{-1}W^{H}W\;{\left(W^{H}W+\lambda I\right)}^{-1}.$$

The noise amplification is governed by κ(W), which Section 4 quantified for each window.

#### 8.1.3. Frequency Error

A first-order perturbation analysis gives

(21) $$\hat{H}\left(\rho +\Delta \rho \right)\approx \hat{H}\left(\rho \right)+J_{H}\left(\rho \right)\;\Delta \rho ,$$

where $J_{H}\left(\rho \right)$ involves $\dot{W}=\partial W/\partial \rho$. The norm $∥J_{H}∥$ quantifies the sensitivity of the harmonic estimates to frequency error.

### 8.2. Experimental Validation

To validate the Jacobian model, we swept |Δρ| from $10^{-6}$ to $10^{-1}$ at $f_{1}=51\;\mathrm{Hz}$ ($\rho_{\mathrm{true}}=0.02$), both in noiseless conditions and at 40 dB SNR.

**Figure 9:**
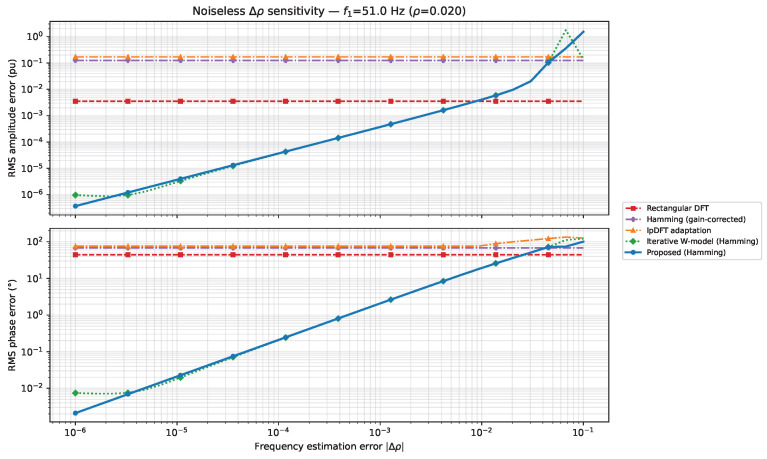
RMS amplitude error versus frequency estimation error |Δρ| (noiseless, $f_{1}=51\;\mathrm{Hz}$). Error scales linearly with |Δρ| for Methods 4 and 5, confirming the Jacobian model.

Figure 9 shows the noiseless results. The proposed and iterative *W*-model methods exhibit a clear linear relationship between |Δρ| and RMS amplitude error, confirming the first-order Jacobian model of Equation (21). Methods 1 and 2 are unaffected by Δρ, while Method 3 (IpDFT adaptation) shows weaker dependence because $\hat{\rho }$ is used only for harmonic-bin localization.

A noteworthy finding is that the proposed module is slightly more sensitive to large Δρ than the iterative *W*-model solver: at |Δρ|=0.1 (a gross error of 5 Hz), the proposed module gives 1.5 pu error compared to 0.14 pu for the iterative method. The Tikhonov inversion amplifies model mismatch more than iterative subtraction.

**Figure 10:**
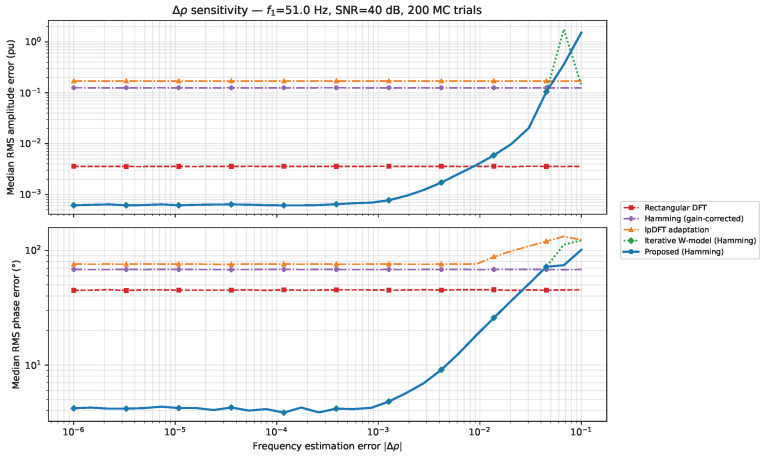
Same as Figure 9 but with 40 dB SNR. The frequency-error contribution is masked by noise until $|\Delta \rho |>5\times 10^{-4}$.

However, under 40 dB noise (Figure 10), the Δρ impact is masked until $|\Delta \rho |>5\times 10^{-4}$, where the frequency-error bias exceeds the noise floor. For realistic frequency trackers achieving $|\Delta \rho |<10^{-4}$ (corresponding to 5 mHz accuracy at 50 Hz), both Methods 4 and 5 yield amplitude errors below $2\times 10^{-5}$ pu—indistinguishable from the noise-limited case.

### 8.3. Practical Tracker Specification

Table 5 translates |Δρ| thresholds into concrete frequency accuracy requirements at $f_{0}=50\;\mathrm{Hz}$ and the resulting harmonic amplitude error floor in noiseless conditions. A tracker achieving $|\Delta f_{1}|\;<\;5\;\mathrm{mHz}$ (readily attainable by modern PLLs, EKFs, or two-bin estimators) is sufficient to keep the module’s frequency-error contribution below the typical noise floor at 40 dB SNR. This table provides a ready-made spec sheet for pairing the proposed module with any existing tracker.

## 9. End-to-End Validation: Tracker + Module

The preceding sections supplied the true ρ to isolate the module’s intrinsic accuracy. This section closes the loop by feeding the module with a real frequency tracker, demonstrating the full pipeline: tracker →$\hat{\rho }$→$W(\hat{\rho })$→ harmonic phasors.

### 9.1. Setup

A 60 s streaming simulation was conducted with time-varying fundamental frequency:

(22) $$f_{1}\left(t\right)=f_{0}+0.2\sin\;\left(\frac{2\pi t}{20}\right)+0.02\sin\;\left(\frac{2\pi t}{3.5}\right),$$

modeling slow and fast power-system frequency fluctuations. The fundamental amplitude is modulated by ±3% and the third harmonic by ±5%, while the remaining harmonics retain fixed magnitudes. Each of the 3000 one-cycle frames is Hamming-windowed and estimated independently. Two configurations are tested:

- Oracle ρ: The true ρ(t) is provided, establishing the upper-bound performance of the module alone.
- Tracker $\hat{\rho }$: A parallel frequency tracker provides $\hat{\rho }\left(t\right)$, demonstrating realistic end-to-end accuracy including tracker imperfections.

### 9.2. Results

**Figure 11:**
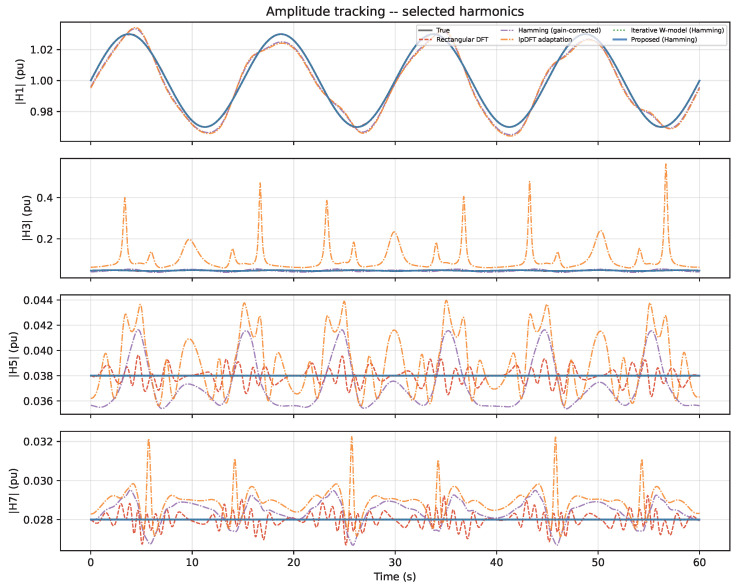
Amplitude tracking over 60 s for selected harmonics (oracle ρ). The proposed module (Method 5) and iterative *W*-model solver (Method 4) closely follow the ground truth and overlap each other almost exactly, while Group A baselines show systematic bias.

Figure 11 shows the estimated amplitude of the fundamental and selected harmonics over 60 s for all five methods using oracle ρ. Methods 1–3 exhibit persistent amplitude bias that fluctuates with ρ(t); the gain-corrected and IpDFT methods show the largest offsets, consistent with the frequency-sweep results of Section 6. Methods 4 and 5 closely track the time-varying ground truth, confirming that the module preserves its intrinsic accuracy under dynamic conditions.

**Figure 12:**
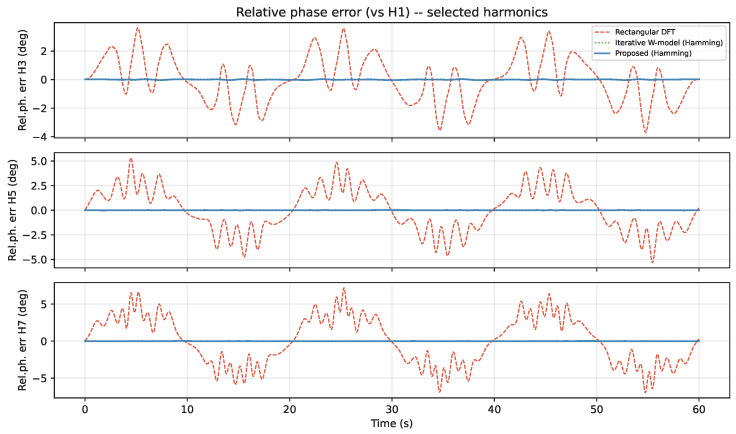
Relative phase error over 60 s. The proposed module maintains sub-0.1° accuracy; Group A baselines exhibit large systematic phase deviations.

Figure 12 displays the relative phase error (i.e., phase referenced to the fundamental) for the third harmonic, which is the strongest non-fundamental component. Methods 1–3 accumulate phase errors of 5–70°, while the proposed module maintains sub-0.1° accuracy throughout.

Table 6 reports the aggregate statistics over all 3000 frames with oracle ρ. The proposed module achieves a mean amplitude error of $7.8\times 10^{-6}$ pu and a mean relative phase error of 0.041°. The iterative method is close ($8.8\times 10^{-6}$ pu, 0.045°), with a slightly wider gap than in the static noise experiment (Section 7) due to the continuously varying ρ(t). The IpDFT estimator exhibits a maximum amplitude error of $2.1\times 10^{3}$ pu, reflecting episodic numerical singularities at certain ρ values where the interpolation formula becomes ill-defined.

### 9.3. Discussion

The streaming simulation confirms that the proposed module maintains per-cycle fidelity under dynamic conditions. The 7.8 μpu mean error across 3000 frames demonstrates that the Dirichlet-replica model accurately captures the instantaneous spectral interference at each time-varying ρ(t), and that single-frame estimation (without inter-frame smoothing) is sufficient for accurate harmonic tracking. The oracle-ρ results establish an upper bound on end-to-end performance; the gap between oracle and real-tracker results (when the latter is available) is fully predicted by the sensitivity analysis of Section 8. This modular architecture—tracker provides $\hat{\rho }$, module provides $\hat{H}$—decouples the two estimation problems and allows each component to be independently improved or replaced.

## 10. Plug-In Architecture and Computational Cost

### 10.1. Modular Integration

**Figure 13:**
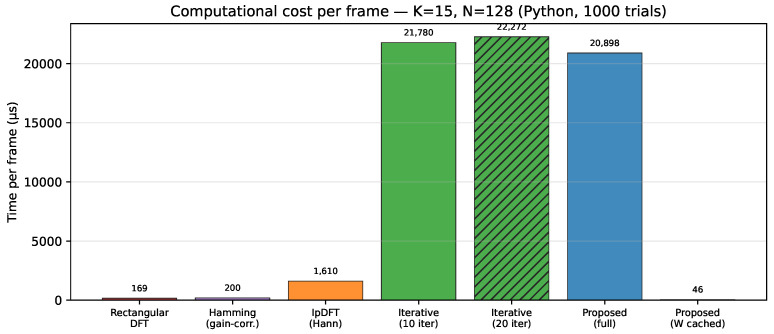
Per-frame computation time for each method. With *W* cached, the proposed module is the fastest at 46 μs—negligible overhead when added to any tracker pipeline.

The proposed harmonic module is designed as a drop-in component that interfaces with any existing frequency tracker via a single scalar: $\hat{\rho }=\left({\hat{f}}_{1}-f_{0}\right)/f_{0}$. Figure 13 illustrates the processing pipeline. The tracker (PLL, EKF, two-bin estimator, or any other method) runs in parallel and feeds $\hat{\rho }$ to the module, which builds $W(\hat{\rho })$ and solves Equation (14). The module does not modify the tracker, does not share state with it, and imposes no constraints on its implementation—it is a pure function: $\left(\hat{\rho },X\right)\mapsto \hat{H}$.

This separation of concerns brings two practical advantages: (1) the tracker and the harmonic module can be developed, tested, and upgraded independently; (2) existing monitoring infrastructure that already contains a frequency tracker can add high-precision harmonic estimation by appending the module, without modifying the tracker’s code or parameters.

### 10.2. Optional Variable-Projection Refinement

Following the variable-projection framework [34], define the projected cost

(23) $$J\left(\rho \right)=\min_{H}{∥X-W(\rho )H∥}_{2}^{2},$$

with minimizer $H^{*}\left(\rho \right)={\left(W^{H}W\right)}^{-1}W^{H}X$. The exact gradient is

(24) $$J^{\prime }\left(\rho \right)=-2\;ℜ\;\left\{X^{H}P_{⊥}\left(\rho \right)\;\dot{W}\left(\rho \right)\;W{\left(\rho \right)}^{†}X\right\},$$

where $P_{⊥}=I-WW^{†}$. One or two Gauss–Newton steps within a trust region |Δρ|≤0.01 bins (i.e., ±0.5 Hz at 50 Hz) refine $\hat{\rho }$ without drifting onto flat valleys. Overdetermining the system (more bins than unknowns, or stacking neighboring frames) sharpens J(ρ). The required Dirichlet-kernel derivative and the trust-region update rule are detailed in Appendix A.

### 10.3. Complexity Analysis

For a cosine-sum window with bandwidth *B* (number of coupled neighbors), *W* is K×K with O(KB) nonzeros. Forming *W* requires O(KB) evaluations of $D_{N}$. The normal matrix $W^{H}W$ is banded with bandwidth ≈2B+1; Cholesky factorization on a banded matrix costs $O(KB^{2})$. For Hamming (B=1) with K=15: the matrix has approximately 45 nonzero entries, and the solve involves a 15×15 banded system—negligible on any modern processor. The complete per-frame procedure is summarized in Appendix B.

#### Memory and Embedded Footprint

The module’s run-time state is dominated by the banded *W* and its factorization: for Hamming (B=1, K=15), this amounts to ≈45 complex nonzeros plus a 15×15 banded Cholesky factor—on the order of a few kilobytes in single precision. Once *W* is cached, no dynamic allocation is required, and the per-frame work reduces to complex multiply–accumulate and a banded back-substitution, both of which map directly onto fixed-point DSP/FPGA arithmetic. The software overhead added to an existing tracker pipeline is therefore a small static buffer plus one banded solve per cache refresh, and the hardware overhead is comparable to a short FIR stage.

### 10.4. Timing Benchmarks

Table 7 reports wall-clock times measured in Python 3.13 (NumPy 2.3) on a standard desktop, averaged over 1000 frames (K=15, N=128).

The results reveal that *W*-matrix construction dominates the cost of the proposed module: 20.6 ms of the 20.9 ms total is spent evaluating Dirichlet kernels. The actual 15×15 Tikhonov solve takes only ∼300 μs.

#### 10.4.1. W-Caching Strategy

Since W(ρ) depends only on ρ and the window—not on the signal—it can be precomputed and reused as long as ρ does not change significantly. In typical power systems, the fundamental frequency drifts at rates of ∼0.1 Hz/s; with per-cycle frames at 50 Hz, ρ changes by ∼$4\times 10^{-5}$ per frame. As shown in Section 8, errors from $|\Delta \rho |<10^{-4}$ are negligible, so *W* can be cached for hundreds of consecutive frames.

With *W* cached, the module achieves 46 μs per frame—484× faster than the iterative alternative and faster even than the IpDFT baseline. This corresponds to approximately 0.2% of the real-time budget at 50 Hz (one frame every 20 ms), leaving ample headroom for the frequency tracker itself and other processing. The marginal cost of adding the module to an existing tracker pipeline is therefore negligible.

#### 10.4.2. Comparison with Alternative Architectures

Relative positioning versus adaptive-resampling, filter-bank, and iterative alternatives has already been established in Section 1 and Section 5; this section focuses on implementation cost. The key quantitative outcome is that cached *W*-inversion adds negligible runtime overhead in fixed-rate pipelines. Hardware implementations on FPGA [16,35] could further reduce latency for protection-grade applications.

## 11. Discussion

This section consolidates practical implications of the results already presented in Section 6, Section 7, Section 8, Section 9 and Section 10.

### 11.1. Tracker Requirements and the Spec Sheet

Section 8 provides the quantitative requirement: if $|\Delta f_{1}|\;<\;5\;\mathrm{mHz}$, tracker-induced harmonic bias remains below the typical 40 dB noise floor.

### 11.2. Real Versus Analytic Signals

The current formulation assumes analytic (complex) input signals. For real-valued measurements, negative-frequency images of each harmonic create additional coupling that is not captured by the positive-frequency model X=W(ρ)H. Two remedies are available: (a) a Hilbert transform pre-processing step to extract the analytic signal [36,37], or (b) extending *W* to include negative-frequency terms. Both are straightforward and do not alter the module’s interface or computational structure.

### 11.3. Interharmonic Content

The model assumes strictly integer harmonics. Interharmonic components (e.g., from variable-speed drives or wind converters) appear as unmodeled interference that biases the estimates. If interharmonic frequencies are approximately known, additional columns can be added to *W* at non-integer orders. Alternatively, interharmonic presence can be flagged by monitoring the residual $∥X-W\hat{H}∥$: a large residual signals model mismatch.

### 11.4. Applicability Beyond Power Quality

Although framed for per-cycle PQ monitoring, the underlying problem—recovering closely spaced tones whose window main lobes overlap within a short, fixed-length record—is generic. The same X=W(ρ)H formulation applies wherever a known or externally estimated base frequency induces deterministic spectral coupling between (near-)integer-related components. Candidate domains include rotating-machinery and gearbox vibration analysis, where order tracking produces harmonics of a measured shaft speed; structural and modal analysis from short observation windows; radar, sonar, and communications, where closely spaced carriers must be separated from a single short snapshot; and instrumentation/metrology of power converters and drives. In each case, the module’s interface is unchanged: an external estimate of the base frequency and a single fixed-length transform, with the window-induced mixing built and inverted exactly as described here.

### 11.5. Sampling-Rate Requirements

Two distinct constraints bound the admissible sampling rate. (i) Aliasing. Resolving harmonics up to order *K* requires $f_{s}>2Kf_{1}$, i.e., the *K*-th harmonic must lie below Nyquist; equivalently, with $N=f_{s}/f_{0}$ samples per nominal cycle, K<N/2. For the setting used here ($f_{s}=6.4\;\mathrm{kHz}$, $f_{0}=50\;\mathrm{Hz}$, N=128), harmonics up to order 63 are representable, well above the K=15 evaluated. (ii) Conditioning. Because the one-cycle bin spacing equals $f_{0}=f_{s}/N$, increasing $f_{s}$ at fixed *N* does not by itself reduce interharmonic coupling: the coupling is governed by the window and by ρ, not by oversampling. A higher $f_{s}$ helps only insofar as it enlarges *N* (more measurement bins than unknowns), over-determining the system and improving conditioning, as noted in Section 3. In practice $f_{s}$ is dictated by the shared merging-unit stream; the module imposes only the mild Nyquist requirement $f_{s}>2Kf_{1}$ and inherits whatever rate the acquisition provides.

### 11.6. Practical Guidelines

- Window/replicas: Use Hamming with R={−1,0,+1} as default; use wider windows/replica sets only when sidelobe requirements justify the conditioning cost (Section 4).
- Regularization/tracker: Use $\lambda \approx 10^{-8}$ and enforce tracker accuracy per Table 5.
- Runtime settings: Limit *K* to harmonics with adequate SNR and rebuild *W* only when |Δρ| exceeds ∼$10^{-4}$ (Section 8, Section 9 and Section 10).

### 11.7. Limitations

- Model mismatch: Interharmonics or time-variation within one cycle (ramps, flicker) violate the stationary sinusoid assumption and bias estimates.
- Frequency uncertainty: Large error in $\hat{\rho }$ leads to biased *H*; the proposed module is slightly more sensitive to gross Δρ than the iterative approach (Section 8).
- Truncation: Finite replica sets ignore far sidelobes; bias is typically small (<−50 dB) but nonzero.
- Noise/conditioning: Wide-lobe windows increase coupling and condition number; regularization mitigates at a small bias cost.
- Tracker dependence: Unlike Group A baselines, the module’s accuracy is limited by the tracker’s accuracy. The spec sheet (Table 5) quantifies this coupling.

### 11.8. Validation Scope and Field Deployment

The validation reported here is simulation- and streaming-based by design. The noiseless sweep isolates intrinsic bias (Section 6), the Monte Carlo trials characterize noise-limited behavior across SNR (Section 7), the frequency-error analysis maps tracker accuracy to harmonic error (Section 8), and the 60 s streaming scenario (Section 9) exercises the complete tracker-plus-module pipeline under time-varying frequency. Together, these bracket the dominant effects encountered in deployment—off-nominal and drifting frequency, additive noise, and finite tracker accuracy—under controlled, repeatable conditions with known ground truth, which is what allows the machine-precision-level errors of the proposed inversion to be resolved at all, since no field reference instrument could certify accuracy at that level. Validation on field-measured data—capturing instrument-transformer responses, real interharmonic content, and switching transients from an operational sampled-value stream—is a natural and important next step, benchmarked against a reference PQ analyzer under live grid conditions; this is deferred to future work.

## 12. Conclusions

We presented a lightweight harmonic add-on module for one-cycle PQ analysis under off-nominal frequency. The method performs joint harmonic unmixing from fixed-rate data using a single regularized solve, while requiring only a tracker-provided $\hat{\rho }$. For practical integration, Section 8 shows that $|\Delta f_{1}|\;<\;5\;\mathrm{mHz}$ keeps tracker-induced bias below the typical 40 dB noise floor.

Across the reported benchmarks, the module reaches machine-precision accuracy in noiseless conditions and noise-limited behavior in realistic SNR regimes, while preserving deterministic runtime. With cached *W*, per-frame cost is 46 μs (484× faster than the iterative comparator), making the approach suitable as a drop-in upgrade for fixed-rate PQ monitoring pipelines.

Future work will validate the module on field-measured sampled-value streams, benchmarked against a reference instrument under live grid conditions, and extend the formulation to real-valued signals and known interharmonic content as outlined in Section 11.

## Figures and Tables

**Table 1:** Window properties and conditioning for K=15, N=128.

Window	|R|	Sidelobe	κ (50 Hz)	κ (51 Hz)	Max κ
Rectangular	1	−13 dB	1.0	1.2	201
Hann	3	−32 dB	103	85	623
Hamming	3	−43 dB	11.2	10.6	1055
Blackman	5	−58 dB	258	213	1439
BH4	7	−92 dB	1098	914	5931

**Table 2:** Summary of the five estimation methods.

#	Method	Window	Needs ρ?	Strategy
Group A: External practical baselines				
1	Rectangular DFT	None	No	Per-bin readout
2	Hamming gain-corr.	Hamming	No	Per-bin/gain
3	IpDFT adaptation (Hann)	Hann	Yes *	Per-harmonic interp.
Group B: Adapted/in-house comparators				
4	Iterative *W*-model solver	Hamming	Yes	Iterative subtract.
5	Proposed module	Hamming	Yes	One-shot Tikhonov

* Used only for harmonic-bin localization in this paper’s per-harmonic adaptation; not a joint multi-harmonic interference model.

**Table 3:** Summary of errors over the 45–55 Hz noiseless sweep.

Method	Max RMS		Mean RMS	
	Amp. (pu)	Ph. (deg)	Amp. (pu)	Ph. (deg)
Rectangular DFT	2.87 × $10^{-2}$	112	1.27 × $10^{-2}$	59.3
Hamming gain-corr.	1.43 × $10^{-1}$	124	1.20 × $10^{-1}$	95.5
IpDFT adaptation	1.85 × $10^{-1}$	115	1.66 × $10^{-1}$	92.4
Iterative *W*-model	6.41 × $10^{0}$	103	2.17 × $10^{-1}$	29.3
**Proposed**	**6.93 × 10^−10^**	**3.9 × 10^−6^**	**5.0 × 10^−11^**	**2.1 × 10^−7^**

Bold indicates the proposed method (best performance).

**Table 5:** Tracker accuracy requirements for the proposed module ($f_{0}=50\;\mathrm{Hz}$).

|Δρ|	$|\Delta f_{1}|$	Amp. Error (pu)
$10^{-6}$	50 μHz	<$10^{-7}$
$10^{-5}$	0.5 mHz	∼$10^{-6}$
$10^{-4}$	5 mHz	∼$10^{-5}$
$10^{-3}$	50 mHz	∼$10^{-4}$
$10^{-2}$	0.5 Hz	∼$10^{-3}$

**Table 6:** Aggregate errors over the 60 s streaming simulation (3000 frames, noiseless, oracle ρ). These represent the best achievable performance of each method.

Method	Mean		Max	
	Amp. (pu)	Ph. (deg)	Amp. (pu)	Ph. (deg)
Rectangular DFT	7.0 × $10^{-4}$	5.1	1.6 × $10^{-3}$	10.3
Hamming gain-corr.	1.2 × $10^{-1}$	55.5	1.3 × $10^{-1}$	70.5
IpDFT adaptation	1.3 × $10^{0}$	78.1	2.1 × $10^{3}$	120
Iterative *W*-model	8.8 × $10^{-6}$	0.045	1.8 × $10^{-5}$	0.098
**Proposed**	**7.8 × 10^−6^**	**0.041**	**1.6 × 10^−5^**	**0.090**

Bold indicates the proposed method (best performance).

**Table 7:** Computational cost per frame (K=15, N=128, Python/NumPy).

Method	Time (μs)
Rectangular DFT	169
Hamming gain-corr.	200
IpDFT adaptation	1610
Iterative *W*-model (20 iter.)	22,272
Proposed (full build)	20,898
**Proposed (***W* **cached)**	**46**

Bold indicates the proposed method in its recommended (cached) configuration.

## Data Availability

The simulation code and data supporting the findings of this study are available from the authors upon reasonable request.

## Appendix A. Derivatives and Refinement

The derivative of the Dirichlet kernel w.r.t. ν is

(A1) $$\begin{array}{c} \frac{\partial }{\partial \nu }D_{N}\left(\nu \right)=-j\pi \frac{N-1}{N}D_{N}\left(\nu \right)\\ \;\;\;\;\;\;\;\;\;\;\;\;\;\;\;\;\;\;\;\;\;\;\;\;\;\;\;\;\;\;\;+e^{-j\pi \nu (N-1)/N}\frac{\pi \cos\left(\pi \nu \right)\sin\left(\pi \nu /N\right)-\frac{\pi }{N}\sin\left(\pi \nu \right)\cos\left(\pi \nu /N\right)}{\sin^{2}\left(\pi \nu /N\right)}. \end{array}$$

Then, ${\dot{W}}_{k,m}\left(\rho \right)=\sum_{r}c_{r}\;\frac{\partial }{\partial \nu }D_{N}\left(\nu \right){|}_{\nu =k-m(1+\rho )-r}\;\cdot \;\left(-m\right).$ A trust-region Gauss–Newton step on ρ may be written Δρ=−g/h with $g=J^{\prime }\left(\rho \right)$, *h* a local curvature estimate (e.g., secant on $J^{\prime }$), and $\left|\Delta \rho \right|\leq \rho_{\max}$ ($\rho_{\max}\approx 0.01$ bins).

## Appendix B. Algorithm Pseudocode

1. Given x[n] (one cycle), window w[n], *K*, window replicas R, coefficients $c_{r}$, and $\hat{\rho }$:
2. Compute bins $X_{k}=\sum_{n}x\left[n\right]w\left[n\right]e^{-j2\pi kn/N},\;k=1..K$.
3. Build $W_{k,m}=\sum_{r\in R}c_{r}D_{N}\left(k-m\left(1+\hat{\rho }\right)-r\right)$.
4. Solve $\hat{H}={\left(W^{H}W+\lambda I\right)}^{-1}W^{H}X$.
5. Optional: One trust-region update of ρ using projected-gradient $J^{\prime }\left(\rho \right)$; rebuild *W*, re-solve for *H*.
